# Commentary: Retrieval practice protects memory against acute stress

**DOI:** 10.3389/fnbeh.2017.00048

**Published:** 2017-03-14

**Authors:** Oliver T. Wolf, Annette Kluge

**Affiliations:** ^1^Department of Cognitive Psychology, Faculty of Psychology, Ruhr University BochumBochum, Germany; ^2^Department of Work and Organizational Psychology, Faculty of Psychology, Ruhr University BochumBochum, Germany

**Keywords:** stress, psychological, stress, physiological, memory retrival, anxiety disorders, work related stress

Nineteen years ago the impairing impact of stress on memory retrieval was observed in rats (de Quervain et al., 1998). Years later this effect was demonstrated in human participants (Kuhlmann et al., 2005). This phenomenon has been replicated repeatedly (e.g., Buchanan et al., 2006; Smeets, 2011) even though, of course, non-significant effects and even the reversed pattern were reported (e.g., Wolf et al., 2002; Hupbach and Fieman, 2012). These non-replications may reflect a more moderate stress response and/or particularities of the learning material used in the studies (for reviews see Cadle and Zoladz, 2015; Gagnon and Wagner, 2016; Wolf, 2017). Studies in rodents revealed that this effect is caused by an interaction of a noradrenergic signal induced by the first response wave (the sympathetic nervous system) and a glucocorticoid signal induced by the second response wave (the hypothalamus-pituitary-adrenal (HPA) axis) (Roozendaal et al., 2006). The effect occurs once cortisol concentrations are elevated approx. 20–30 min after stress onset. The impairment lasts 2–3 h, which is longer than the typical stress induced cortisol elevations most likely reflecting non-genomic and genomic effects (Gagnon and Wagner, 2016; Wolf, 2017). Pharmacological studies revealed that blocking glucocorticoid secretion or blocking noradrenergic arousal with a beta blocker prevents the stress effects (de Quervain et al., 1998, 2007).

In a recent paper published in Science, Smith, Floerke and Thomas tested the impact of different learning strategies on this effect (Smith et al., 2016). They used the retrieval practice strategy where participants try to retrieve the recently seen items without feedback. This strategy leads to superior memories in contrast to typical study practice (passive re-reading) approaches (Karpicke and Roediger, 2008; Roediger and Butler, 2011). On a side note it should be mentioned that studies using complex and dynamic learning material for controlling complex technical systems could, however, not observe the superiority of the testing- effect compared to additional practice (Kluge and Frank, 2014). Smith and colleagues reported that retrieval practice lead to superior memory performance. More importantly retrieval practice prevented the impairing effects of stress on memory retrieval observed in the group of participants which learned the items using the classical study practice approach. The study is thus the first to demonstrate that a rehearsal strategy might be able to create memory traces which are less sensitive to stress. Since the retrieval practice group shows an overall better memory performance it remains open whether the missing stress effects reflects better learning in the sense of “stronger memory traces in general are less susceptible to stress” or whether the findings might be related to the specifics of this strategy. Another experimental group using the study practice approach but with more repetitions would have been an option in order to create a group with similar performance to the retrieval practice group resulting from a less optimal passive encoding strategy. Additional methodological concerns come from the missing cortisol assessment, not allowing the demonstration of a successful stress induced HPA activation which is comparable between the two different groups. Future studies are needed to understand the specificity of the observed effects for the retrieval practice strategy and to characterize the underlying endocrine and neural mechanisms.

The possibility to create, at the time of encoding, memory traces which are less susceptible to the impairing effects of stress on memory retrieval can pave the way for new research with substantial promises for the applied areas of psychology. The relevance for the educational setting (schools and universities) are directly obvious. In addition clinical applications are conceivable. Stress has been associated with a return of fear after extinction training in rodent studies (Stockhorst and Antov, 2015; Maren and Holmes, 2016). In patients fear often comes back after successful extinction based therapies when the patients are stressed (Jacobs and Nadel, 1985). Similarly in laboratory based analog studies using conditioning paradigms acute stress lead to a return of the original acquired response (Hamacher-Dang et al., 2013; Raio et al., 2014; Kinner et al., 2016). The current study could stimulate researchers to develop and test psychological methods aimed at creating extinction memory traces which are resistant to stress. Among potential candidates deepened extinction, generalized extinction, gradual extinction (e.g., Shiban et al., 2015), the use of multiple contexts (e.g., Dunsmoor et al., 2014) could be promising candidates (see for review Pittig et al., 2016).

Last but not least the findings are also of relevance to organizational psychology and human factor research. Due to the emerging digitalization of business processes and automation in production, many skills acquired during vocational training face the challenge of long periods of non-use. These infrequently executed skills than need to be recalled and applied in so called non-routine situations which lead to higher levels of stress, due to perceived time and production pressure, alarm flooding and other environmental stressors alike (Kluge et al., 2014). In that respect the findings by Smith et al. are also of relevance for Human Factors applications but need to be validated for more complex learning material. Retrieval practice implemented by means of imaginary practice or symbolic rehearsal could be considered, as in symbolic rehearsal, a person visualizes how to perform a task without actually performing the task (Annett, 1979; Driskell et al., 1994). Results of a meta-analysis by Driskell et al. (1994) showed that the more mental operations the task requires, the more effective imaginary practice is, especially for novice learners.

Taken together the study by Smith and colleagues opens up a new research venue for psychological research aiming at preventing the stress induced retrieval impairment repeatedly observed in the laboratory by modifying the initial acquisition of the memory trace of interest. This line of research would complement the pharmacological approaches successfully tested in the past (de Quervain et al., 1998, 2007). The most effective learning strategy may differ according to the memory domain of interest. This line of research has the potential to help preventing stress induced relapses as well as stress induced errors in the working-environment.

## Author contributions

OW and AK discussed jointly the potential and the limitations of the original manuscript and drafted and wrote the commentary.

## Funding

The research from OW and AK is funded by the German Research Foundation (DFG). KL2207/3-1, KL2207/3-3, WO 733/13-2, and the collaborative research center (SFB) 874.

### Conflict of interest statement

The authors declare that the research was conducted in the absence of any commercial or financial relationships that could be construed as a potential conflict of interest.
